# Supplementary figures and images for: Probenecid inhibits SARS-CoV-2 replication in vivo and in vitro
Source: Sci Rep. 2021 Sep 10;11:18085. doi: 10.1038/s41598-021-97658-w (PMC8433326; doi:10.1038/s41598-021-97658-w)

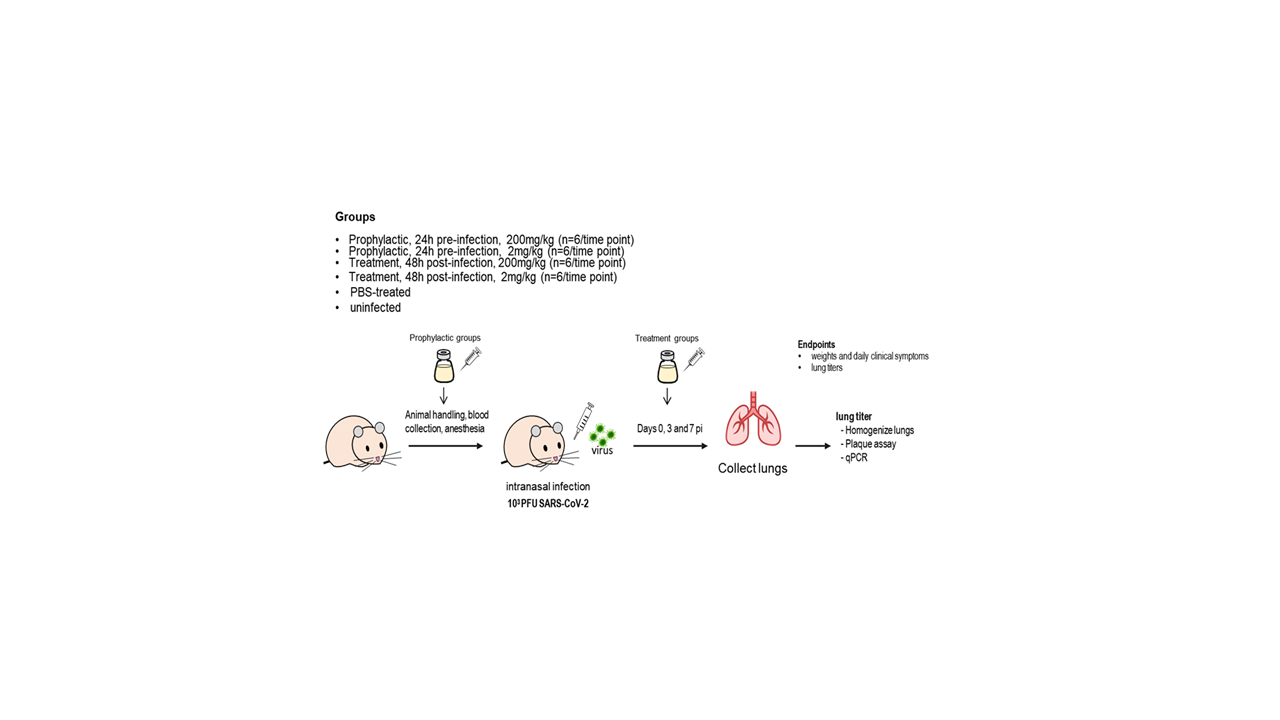

Supplement: Supplementary file 1 — Supplementary Figure 1. [file 41598_2021_97658_MOESM1_ESM.tif]

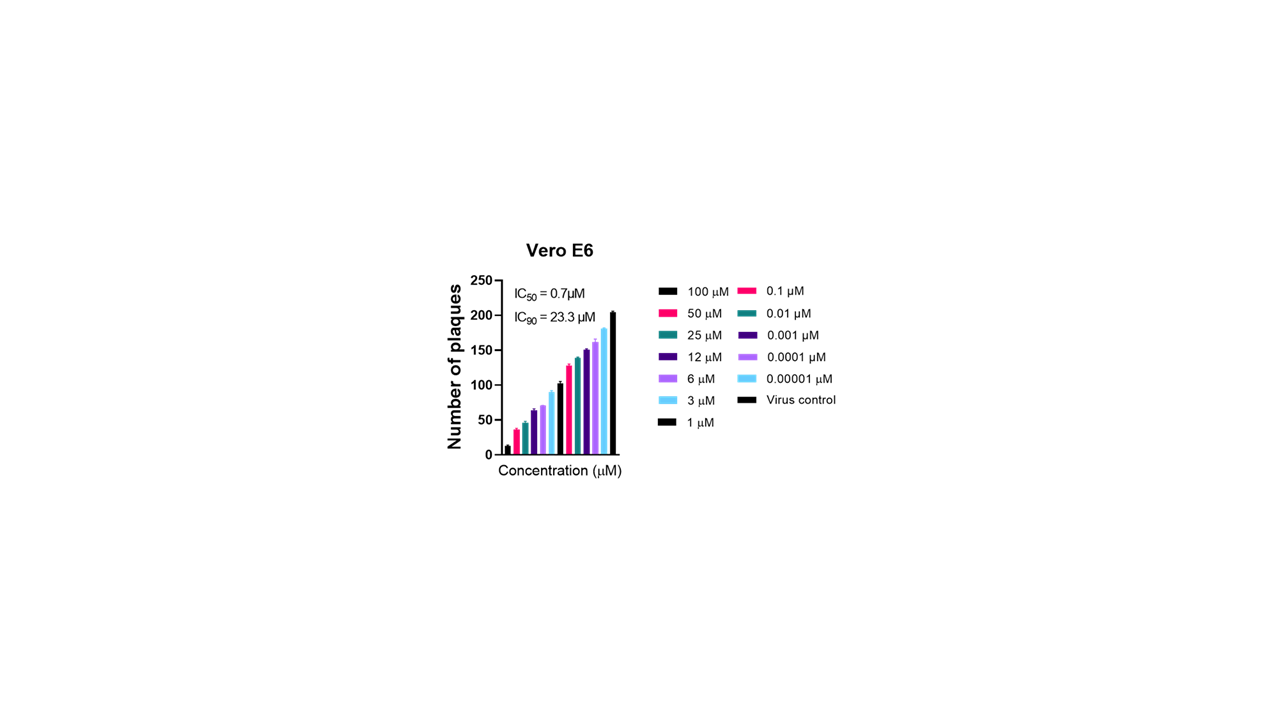

Supplement: Supplementary file 2 — Supplementary Figure 2. [file 41598_2021_97658_MOESM2_ESM.tif]

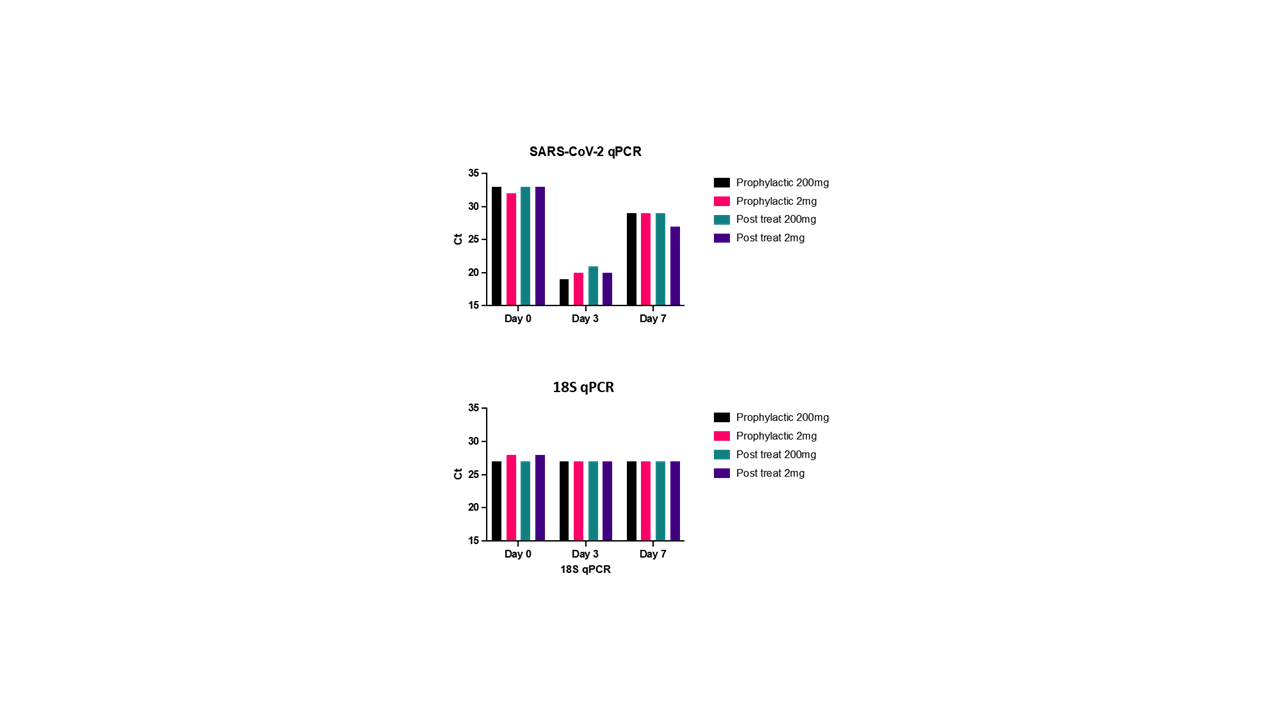

Supplement: Supplementary file 3 — Supplementary Figure 3. [file 41598_2021_97658_MOESM3_ESM.tif]
